# Paracrine Effect of Mesenchymal Stem Cells Derived from Human Adipose Tissue in Bone Regeneration

**DOI:** 10.1371/journal.pone.0107001

**Published:** 2014-09-08

**Authors:** Itali Linero, Orlando Chaparro

**Affiliations:** 1 Faculty of Dentistry, Universidad Nacional de Colombia, Bogotá, Colombia; 2 Faculty of Medicine, Universidad Nacional de Colombia, Bogotá, Colombia; University of Torino, Italy

## Abstract

Mesenchymal stem cell (MSC) transplantation has proved to be a promising strategy in cell therapy and regenerative medicine. Although their mechanism of action is not completely clear, it has been suggested that their therapeutic activity may be mediated by a paracrine effect. The main goal of this study was to evaluate by radiographic, morphometric and histological analysis the ability of mesenchymal stem cells derived from human adipose tissue (Ad-MSC) and their conditioned medium (CM), to repair surgical bone lesions using an in vivo model (rabbit mandibles). The results demonstrated that both, Ad-MSC and CM, induce bone regeneration in surgically created lesions in rabbit's jaws, suggesting that Ad-MSC improve the process of bone regeneration mainly by releasing paracrine factors. The evidence of the paracrine effect of MSC on bone regeneration has a major impact on regenerative medicine, and the use of their CM can address some issues and difficulties related to cell transplants. In particular, CM can be easily stored and transported, and is easier to handle by medical personnel during clinical procedures.

## Introduction

The use of stem cells and particularly Mesenchymal Stem Cells (MSC) in clinical practice has increased considerably in the last decade. During this time, the scientific community has tried to understand their biological mechanisms of action in tissue repair and regeneration and unveil their potential in cell therapy and regenerative medicine [1], [2].

Although MSC were initially isolated from bone marrow, MSC from adipose tissue (Ad-MSC) are emerging as the best option for clinical applications [3]. Since its description in 2001 [4], the data collected so far have show that adipose tissue is an abundant source of MSC, and due to its wide body distribution makes it accessible by minimally invasive methods. These MSC are also easy to isolate and expand in vitro [5], [6].

The initial focus of MSC treatment of musculoskeletal injuries was based on their ability to differentiate into several cell types [1], [7], [8]. In essence, the expectation was that upon implanting or injecting MSC, the cells would colonize and differentiate at the lesion site along the appropriate MSC linage. This mechanism of action of MSC is currently been challenged, changing the current paradigm to extend it to an alternative mechanism called paracrine effect, where MSC secrete biologically active molecules that exert beneficial effects on injured tissues [9] by promoting angiogenesis and tissue regeneration and inhibiting fibrosis, apoptosis and inflammation [10], [11]. It has also been shown that they have neurogenic, neuroprotective and synaptogenic effects [12], [13]. Since the survival and differentiation of MSC at the site of the lesion is limited, it is proposed that paracrine signaling is the primary mechanism of their therapeutic effects [14]. This hypothesis is supported by in vitro and in vivo studies showing that many cell types respond to paracrine signaling from MSC, causing the modulation of a large number of cellular responses, such as survival, proliferation, migration and gene expression [11].

The secretion of bioactive factors is thought to play a critical role in MSC mediated paracrine activity. These factors and cytokines may be collected in what has been called the conditioned medium (CM), which when transplanted into animal models of different diseasses have similar effects to those exerted by the cells, increasing the tissue repair process in acute myocardial infarction [15], [16], wound healing [17], [18] and as a neuroprotective agent [19].

In this study, we evaluated the ability of Ad-MSC and their CM, to repair bone lesions in an in vivo model, using Human Blood Plasma Hydrogels (HBPH) as a delivery system. The gels obtained from human plasma have been used in tissue engineering to provide a minimally invasive, biodegradable and histocompatible scaffold for cell expansion in vitro and cell delivery for implantation in vivo [20]. The results demonstrate that both, Ad-MSC and CM, induce bone regeneration in surgically created defects in rabbit's jaws. To the best of our knowledge, this is the first direct demonstration of the paracrine effect of Ad-MSC in bone regeneration and raises the possibility of using MSC conditioned media as a promising therapeutic alternative.

## Materials and Methods

### Ad-MSC isolation and culture

Adipose tissue samples for the study were obtained from biopsies of approximately 1 cm^3^ from one female individual, 23 years old, Bichat's fat pad, scheduled for maxillofacial surgery, with previous approval and signing of informed consent and with the approval of the ethics committee of the Faculty of Medicine of the Universidad Nacional de Colombia (Act Number 93).

Explants of approximately 0.2 cm in diameter of adipose tissue were planted in plastic 6-well culture plates (Greiner Bio-one), in 2 ml of Dulbecco's Modified Eagle's low glucose medium, (DMEM, Invitrogen), supplemented with 10% fetal bovine serum (FBS, Invitrogen), penicillin 100 U/ml, streptomycin 100 µg/ml (Sigma Aldrich Inc.) and incubated at 37°C in a humid atmosphere with 5% CO_2_. Half of the medium was replaced with fresh medium twice a week, until the cells reached 70–80% confluence.

### Ad-MSC characterization

Ad-MSC were characterized according to the criteria set by the International Society of Cell Therapy [21]. Ad-MSC immunophenotype was evaluated by flow cytometry identifying surface markers CD105, CD34, CD45, CD90, HLA-ABC and HLA-DR and multipotenciality was evaluated by osteogenic and adipogenic in vitro differentiation, as described before [22].

### Preparation and characterization of Conditioned Medium (CM)

Conditioned medium (CM) was obtained from Ad-MSC at passage 6–7. In order to collect the CM, Ad-MSC were first cultured in FBS-DMEM, 100 U/ml penicillin, 100 µg/ml streptomycin and incubated at 37°C in a humid atmosphere with 5% CO_2_. When the cells reached 70–80% of confluence, they were washed twice with 1X Phosphate Buffered Saline (PBS, Invitrogen), incubated in serum-free medium (OPTIMEM, Invitrogen) under hypoxic conditions (2% O_2_), at 37°C in a humid atmosphere with 5% CO_2_ for 24 hours. The media was collected and cleared by 10 minutes centrifugation at 1200 x g; protein concentration was adjusted with OPTIMEM to 100 (CM-1) and 200 µg/ml (CM-2) and sterilized by filtration through to a 220-nm syringe filter (CORNING) and stored at −20°C until used.

Conditioned media were evaluated for the detection of 43 human proteins including cytokines, growth factors, proteases and soluble receptors, using the Human Angiogenesis Antibody Array C1000 (RayBiotech, Norcross, GA, USA), according to the manufacturer's instructions. Briefly, 1 ml of conditioned medium was incubated with arrayed antibody membranes for 2 h at room temperature; membranes were then washed and incubated with the mix of biotin-conjugated antibodies for another 1 h at room temperature. After washing, HRP-conjugated streptavidin was added to the membranes for 1 h at room temperature. The signal was developed with detection buffer, and membranes were exposed to autoradiographic films. Signal optical densities were quantified using the program for digital image processing Image J 1.410 (NIH, USA). Signal densities (pixels), were normalized to total protein concentration and then expressed as Arbitrary Units/µg protein.

### Preparation of Human Blood Plasma Hydrogels (HBPH)

Plasma samples for preparation of HBPH were obtained from venipuncture from the same patient in which the adipose tissue was obtained, previous approval and signing of informed consent and with the approval of the ethics committee of the Faculty of Medicine of the Universidad Nacional de Colombia (Act Number 93).

Preparation and characteristics of HBPH has been described before [22]. Briefly, HBPHs of 250 µl were prepared using human blood plasma (83 µl), 1X PBS (133 µl), Tranexamic Acid (Ropsohn Therapeutics Ltda) 100 mg/ml (1.6 µl), Calcium Chloride 1% (CaCl2, Sigma-Aldrich Inc.) (16.6 µl), and Dulbecco's Modified Eagle's medium low glucose (DMEM, Invitrogen) (16.6 µl). The gelation time from the moment of the addition of CaCl_2_ was 3–5 minutes, approximately. HBPH exhibited a transparent, homogeneous composition, without sediments or turbidity. These features allow microscopic visualization of Ad-MSCs during proliferation and differentiation. Scanning electron microscopy showed that fibrin networks form a three dimensional structure that generates a mesh with interconnected pores, which have an average diameter between 20 to 30 µm [22].

For the preparation of hydrogels with Ad-MSC, 60,000 sixth passage Ad-MSC resuspended in 16.6 µl DMEM were used instead of the 16.6 µl of DMEM in the gel preparation described above. For the preparation of hydrogels with conditioned media, DMEM was replaced by 16.6 µl of CM-1 or CM-2.

### Animal Model

Nineteen adult male New Zealand white rabbits, 3 months old, weighing between 2–3 kg were purchased and maintained at the Central Animal Facility of the Universidad Nacional de Colombia (Bogotá - Colombia). Bilateral and bicortical surgical defects of 10 mm diameter were created in the mandibular angles [23]. Animals were divided in three groups. In the first group, 12 rabbits were implanted with HBPHs with Ad-MSC and with HBPHs without cells on the contralateral side (control side). Subgroups of 4 animals were sacrificed at 15, 30 and 45 days after the surgery. In the second group, 4 rabbits were implanted with HBPHs with Ad-MSC on both sides and sacrificed at 3, 6, 9 and 12 days after the treatment. In the third group, 3 rabbits were treated with hydrogels containing CM-1 on one side and CM-2 on the contralateral side. Animals were sacrificed 45 days after the surgery. After animal sacrifice, surgical specimens were obtained, comprising the initial bone defect with surrounding soft and hard tissue for the corresponding analysis.

The study was in accordance with the National Institutes of Health and National Society of Medical Research guidelines for care and use of laboratory animals and under the approval of the Ethics Committee of the Faculty of Medicine of the Universidad Nacional de Colombia (Act Number 12).

### Surgical Procedure

Rabbits were anaesthetized with an intramuscular injection of xylazine (Rompun, Bayer S.A.) (3 mg/kg) and ketamine (Imalgene, Rhone Merieux) (20 mg/kg). During the surgery, supplemental sedation was administered when needed. Local anesthesia of a 2% Lidocaine with 1∶80,000 adrenaline (Ropsohn Therapeutics Ltda) was given regularly at the site of incision. Intramuscular injection of benzathine penicillin (Genfar) (30,000 U/Kg) was administered an hour prior to the surgery.

**Figure 1:**
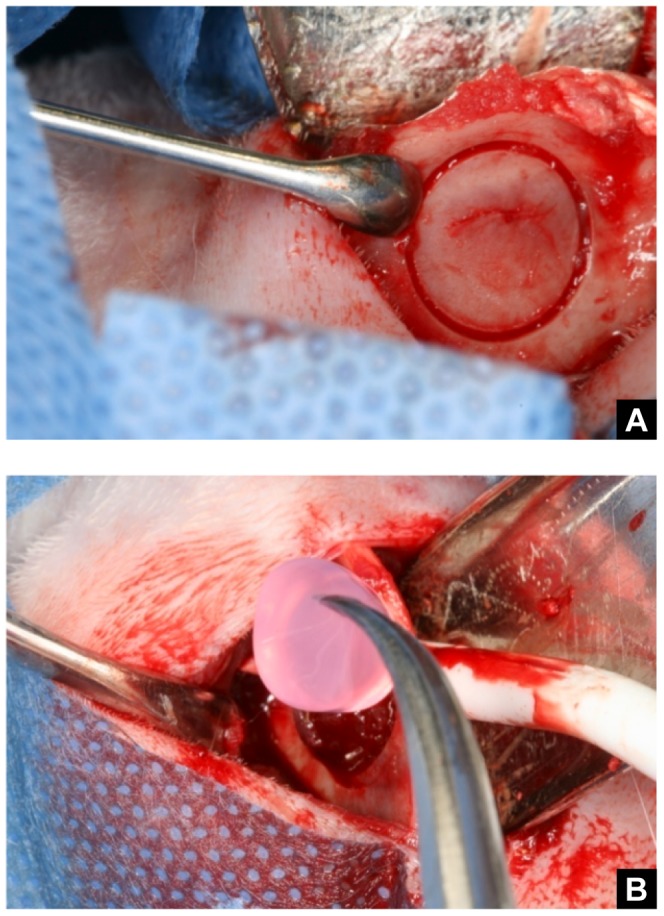
Surgical procedure. **A.** Circular demarcation of bone defect. **B.** Implantation of HBPH.

After shaving the skin and disinfecting the surgical area, a skin incision was made along the inferior border of the mandible in both sides. After exposing the masseter muscle, a sub-periosteal muscle's elevation was made and the muscle was buccally and lingually detached, exposing the mandible angle. A slowly rotating trephine bur (SALVIN) was used to create circular defects 10 mm in diameter in the anterior region to the mandibular angles [23] (Figure 1). The critical size defect, that prevents spontaneous healing during the animal lifetime, in this experimental model is 5 mm [24]. Hydrogels were implanted according the treatment. Soft tissues, including the periosteum, were carefully repositioned and sutured using a 3-0 resorbable suture (Vicryl, Ethicon, Jhonson & Jhonson Company).

### Radiographic Analysis

Analog radiographic equipment (MULTIX, Simens) was used for postoperative lateral skull radiographs and digital radiographic equipment (Veraviewepocs 2D, J Morita) was used for post mortem periapical radiographs. Biplanar radiographs were obtained under standard conditions (42Kv 2.2 mA and 21Kv, respectively). Radiographic images were digitized and analyzed with the program Image J 1.410 (NIH, USA). The total area of regenerated bone tissue was evaluated by measuring the newly formed bone tissue as compared with the initial defect (10 mm diameter).

### Morphometric Analysis

After animal sacrifice, surgical specimens were obtained, comprising the initial bone defect with surrounding soft and hard tissue. Photographs were taken and the percentage of newly formed bone tissue estimated over the time using the Image J 1.410 program (NIH, USA).

### Histological Analysis

Surgical specimens were taken and fixed by immersion in 10% formaldehyde solution (Sigma-Aldrich Inc.) pH 7.4 at 4°C for 72 hours, decalcified in Shandon TBD-1 Rapid Decalcifer (Thermo Fisher Scientific) for 24 hours, dehydrated in ascending series of ethanol (70, 80, 90 and 100%) and transferred to a solution of xylene 100% for 30 minutes. Samples were embedded in paraffin, and 4µm histological sections were stained with hematoxylin-eosin (Thermo Fisher Scientific), blue toluidine (Thermo Fisher Scientific), to identify condroitin sulfate in the cartilage matrix and intramembranous bone, and Masson trichrome (Thermo Fisher Scientific), to observe collagen fibers and calcification process.

To track Ad-MSC, mandibular specimens were fixed in a solution of 10% formaldehyde, pH 7.4 at 4°C for 72 hours, decalcified in EDTA for two months, dehydrated in ascending series of ethanol and transferred to a solution of xylene 100% for 30 minutes. Samples were embedded in paraffin, and 4 µm histological sections obtained for immunohistochemical detection of human ß-2 microglobulin positive cells.

### Immunohistochemistry

To detect implanted human Ad-MSC, 4 µm sections were deparaffinized, incubated with horse serum (Sigma-Aldrich Inc.) treated with anti-human ß-2 microglobulin-HRP primary antibody (1∶200, mouse monoclonal, AbD Serotec) for one hour at room temperature. Histological sections were incubated with biotinylated secondary antibody (1∶300, goat anti-mouse IgG (H + L) BA2000, Vector) for thirty minutes at room temperature and revealed with diaminobenzidine (DAB Plus Substrate Kit, Zymed Lab).

Samples of skin and bone used as controls were obtained from the same patient from which the adipose tissue was obtained, previous approval and signing of informed consent and with the approval of the ethics committee of the Faculty of Medicine of the Universidad Nacional de Colombia.

Human surgical specimens received the same histological and immunohistochemical treatments that rabbit surgical specimens used in this study.

### Statistical Analysis

Differences of the data between groups and between experimental treatments in each group were assessed by General Linear Model's test and t student's test using the Statistical Analysis Software (SAS) version 7.2. The differences were considered to be significant at p≤0.05.

## Results

### Ad-MSC characterization

**Figure 2:**
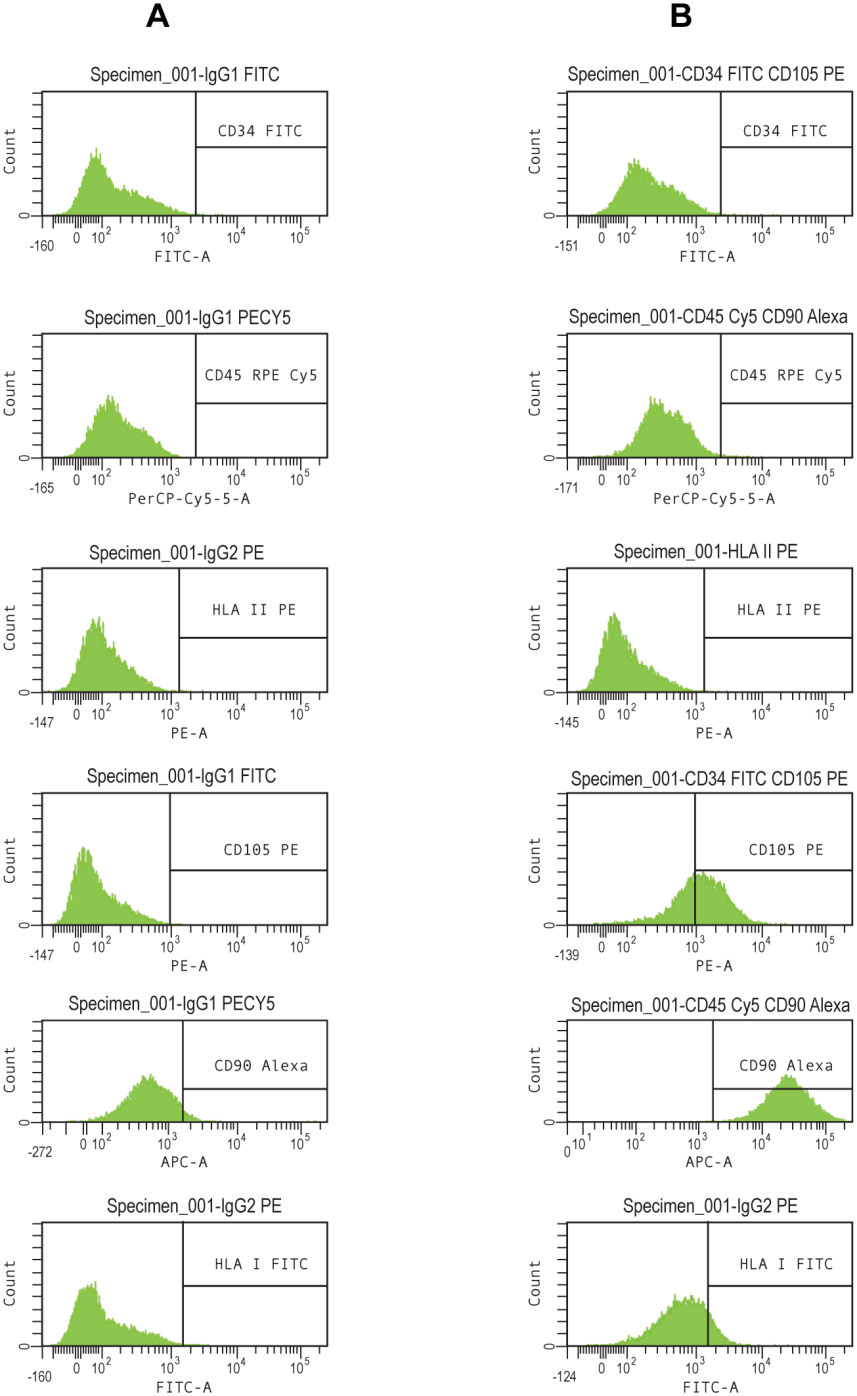
Ad-MSC characterization: Flow Cytometry. Sixth passage, 70% confluence Ad-MSC, were labeled with monoclonal antibodies and analyzed by flow cytometry. **A.** Isotype controls for each of marker. **B.** Ad-MSC labeled with CD34-FITC, CD45-RPECy5, HLA II-RPE, CD105-PE, CD90-Alexa, HLA I-FITC.

**Figure 3:**
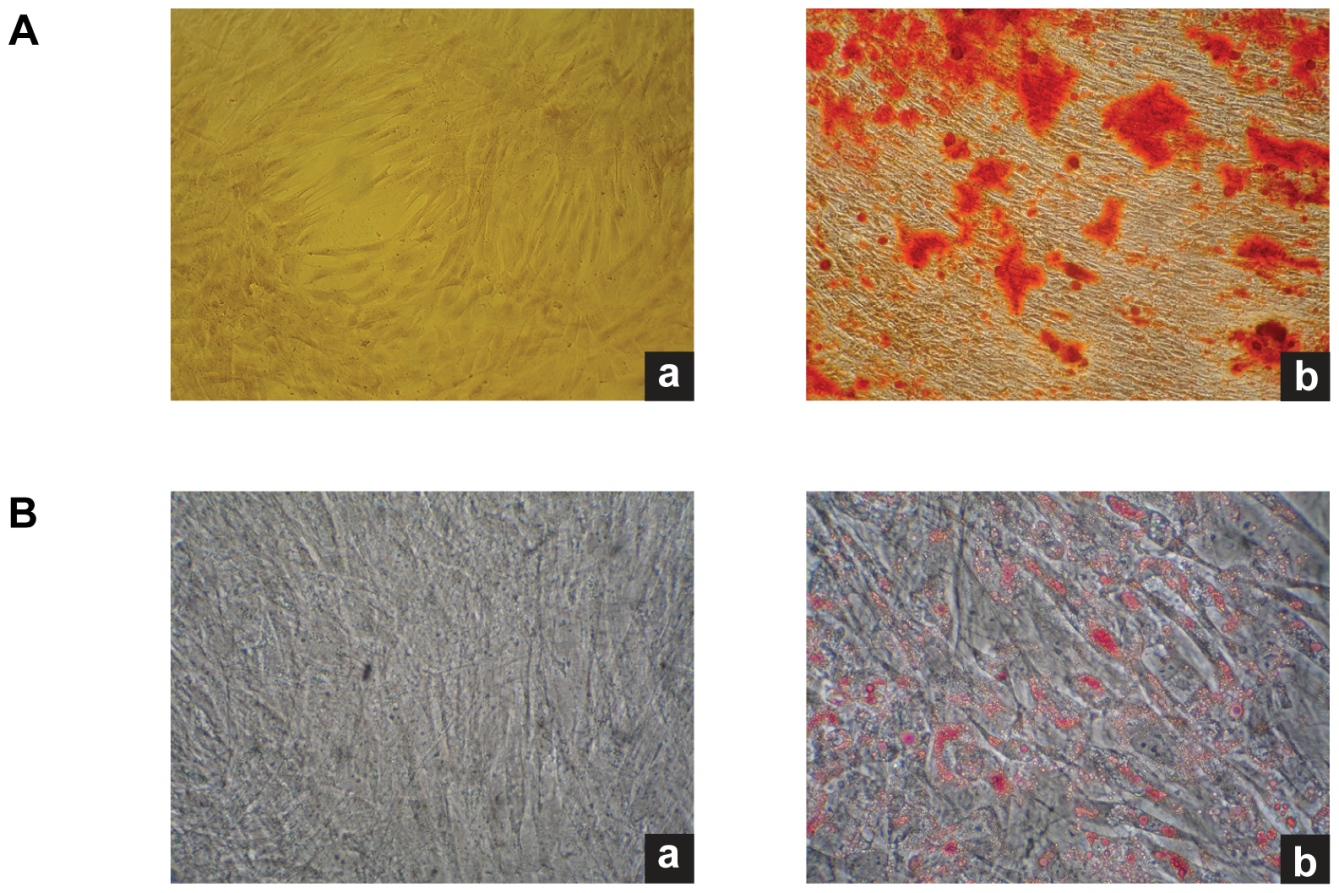
Ad-MSC characterization. **A.** Osteogenic differentiation of Ad-MSC. Osteogenic differentiation was evidenced by the detection of calcium deposits with Alizarin Red staining. **a.** Control Ad-MSCs without osteogenic induction. **b.** Ad-MSC cultured for 3 weeks in osteogenic differentiation medium. **B.** Adipogenic differentiation of Ad-MSC. Adipogenic diferentiation was evidenced by the formation of lipid vacuoles after three weeks of cultivation in adipogenic induction medium. **a.** Control cells without induction. **b.** Lipid vacuoles staining with oil red O. 10× magnification.

The phenotype of Ad-MSC was determined by flow cytometry. Ad-MSC were positive for CD90, CD105, HLA I and negative for CD45, CD34 and HLA II (Figure 2). This immunophenotype is consistent with the parameters established by the International Society for Cellular Therapy [21]. Mineral deposits were evident in Ad-MSC cultured for three weeks in osteogenic differentiation medium, but not in not induced (control) Ad-MSC. Alizarin Red staining was performed to assess the extent of mineralization (Figure 3A). The appearance of lipid vacuoles (rich in triglycerides), was evidenced by positive oil red O staining in Ad-MSC after three weeks of treatment with adipogenic induction medium but not in control Ad-MSC (Figure 3B).

### Secretion of Factors involved in bone regeneration

Using the Human Angiogenesis Antibody Array C1000 (RayBiotech, Norcross, GA, USA), it was demonstrated that Ad-MSC secrete 43 angiogenic factors (cytokines, growth factors, proteases and soluble receptors), and that the secretion was improved when the cells were cultivated in hypoxic conditions as compared with cells cultured in normoxia. From these 43 factors, a group of 11 factors have been reported to be involved in bone regeneration (Table 1). CM from Ad-MSC cultured in hypoxia was then used in further experiments.

**Table 1:** Factors involved in bone regeneration secreted by Ad-MSC cultured under normoxic and hypoxic conditions.

FACTOR	Relative concentration (Arbitrary Units/µg protein)	
	Normoxia	Hypoxia
IL-6	23.8	83.6
VEGF	8.9	19.2
ANGIOGENIN	1.0	7.7
MCP-3	0	11.2
MCP-1	18.9	37.9
IGF-1	5.1	229
TGF - ß	3.7	7.7
PDGF-BB	11.0	23.3
bFGF	10.3	25.9
EGF	7.8	22.7
RANTES	11.2	26.0

The values in the table indicate the relative levels of secretion (Arbitrary Units/µg of protein), of secreted factors produced by Ad-MSC cultured under normoxic and hypoxic conditions. All these factors have been reported to be involved in bone regeneration. IL-6: Interleukin 6, VEGF: Vascular Endotelial Growth Factor, Angiogenin, MCP-3:Monocyte Chemoattractant Protein-3, MCP-1: Monocyte Chemoattractant Protein-1, IGF-1:Insulin Like Growth Factor-1, TGF-ß: Transforming Growth Factor Beta, PDGF-BB: Platelet Derived Growth Factor Isoform BB, bFGF: Basic Fibroblast Growth Factor, EGF: Epidermal Growth Factor, RANTES: Regulated upon Activation Normal T-cell Expressed, and Secreted.

### Bone regeneration by implanting HBPHs with Ad-MSC

#### Radiographic Analysis

**Figure 4:**
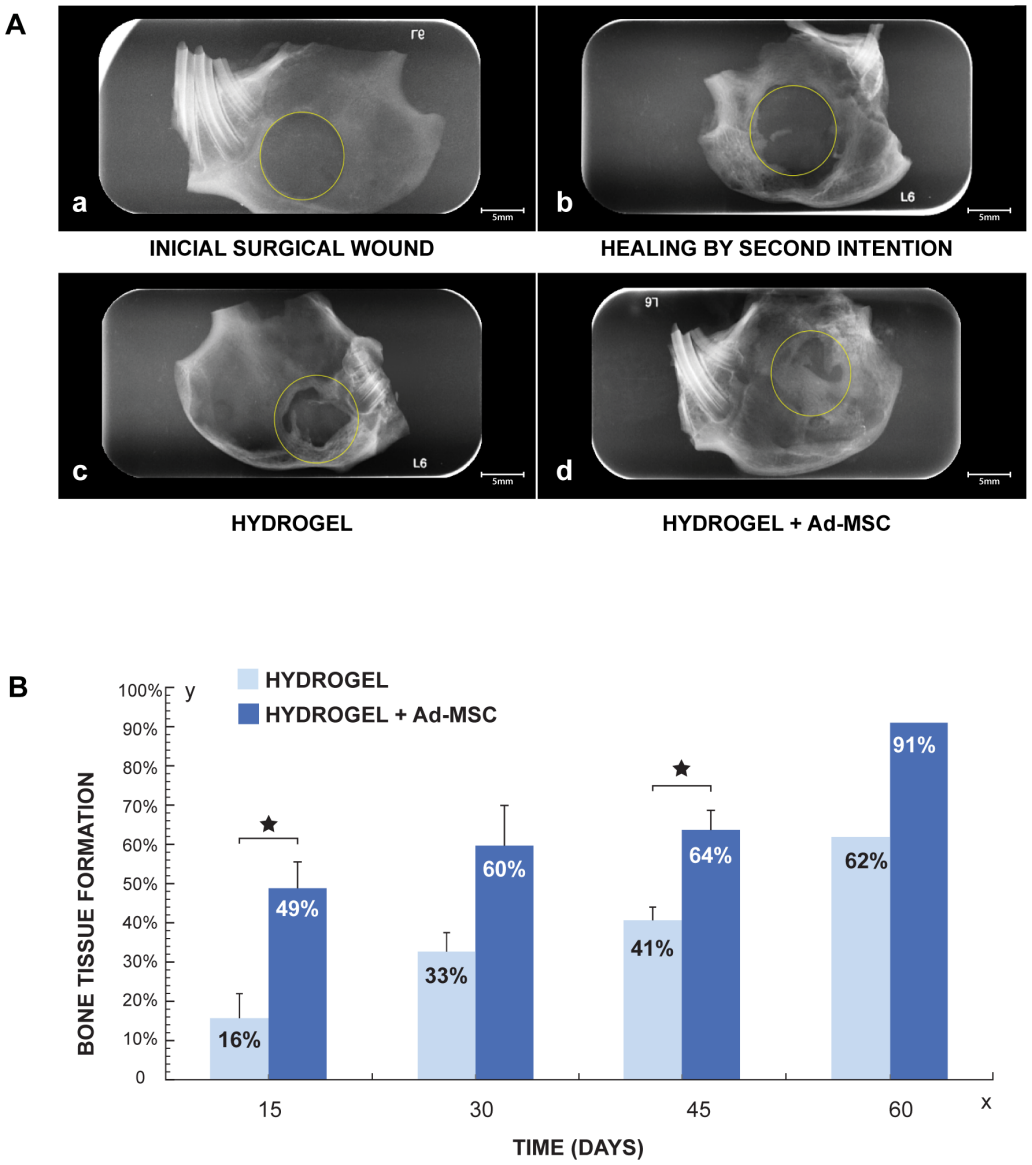
Radiographic Analysis of bone regeneration by implanting HBPHs with Ad-MSC. **A.** Radiographic comparison of bone defects at 45 days with different treatments. **a.** Initial size of surgical wound. **b.** Healing by second intention. **c.** Bone defect treated with hydrogel. **d.** Bone defect treated hydrogel with Ad-MSCs. **B.** Histogram represent the average of newly formed bone tissue at 15, 30, 45 (n = 4) and 60 (n = 1) days after grafting Hydrogel with Ad-MSC (dark blue) and Hydrogel without Ad-MSC (light blue).

The formation of new bone tissue was documented radiographically. Figure 4, shows radiographic comparisons of bone defects at 45 days with different treatments. In areas where surgical defects were allowed to heal by second intention a small radiopaque halo from the edges of bone defects was observed (Figure 4Ab). A larger radiopaque area, from the periphery toward the center of the lesion was observed in bone defects implanted with hydrogel without cells (Figure 4Ac) and a radiopaque area covering more than 70% of the initial bone defect size, was evident in the side where the hydrogel with Ad-MSC was implanted (Figure 4Ad).

Quantitative analysis using Image J1.410 program, showed a more pronounced reduction of bone defects in areas treated with hydrogel plus Ad-MSC as compared with control side. Fifteen days after the surgery, the side treated with Ad-MSC showed a new bone formation area of 49% (+/−13) compared with a 16% (+/−11) in control side. At 30 days, a 60% (+/−15) recovery in the treated side was observed as compared with a 33% (+/−9) of control side and at 45 days the percentage of new tissue in the treated side increased to 64% (+/−7) and in control side to 41% (+/−7). The evaluation on day 60 was performed in a single individual and showed close to 100% closure of the surgical wound (Figure 4B).

#### Morphometric Analysis

**Figure 5:**
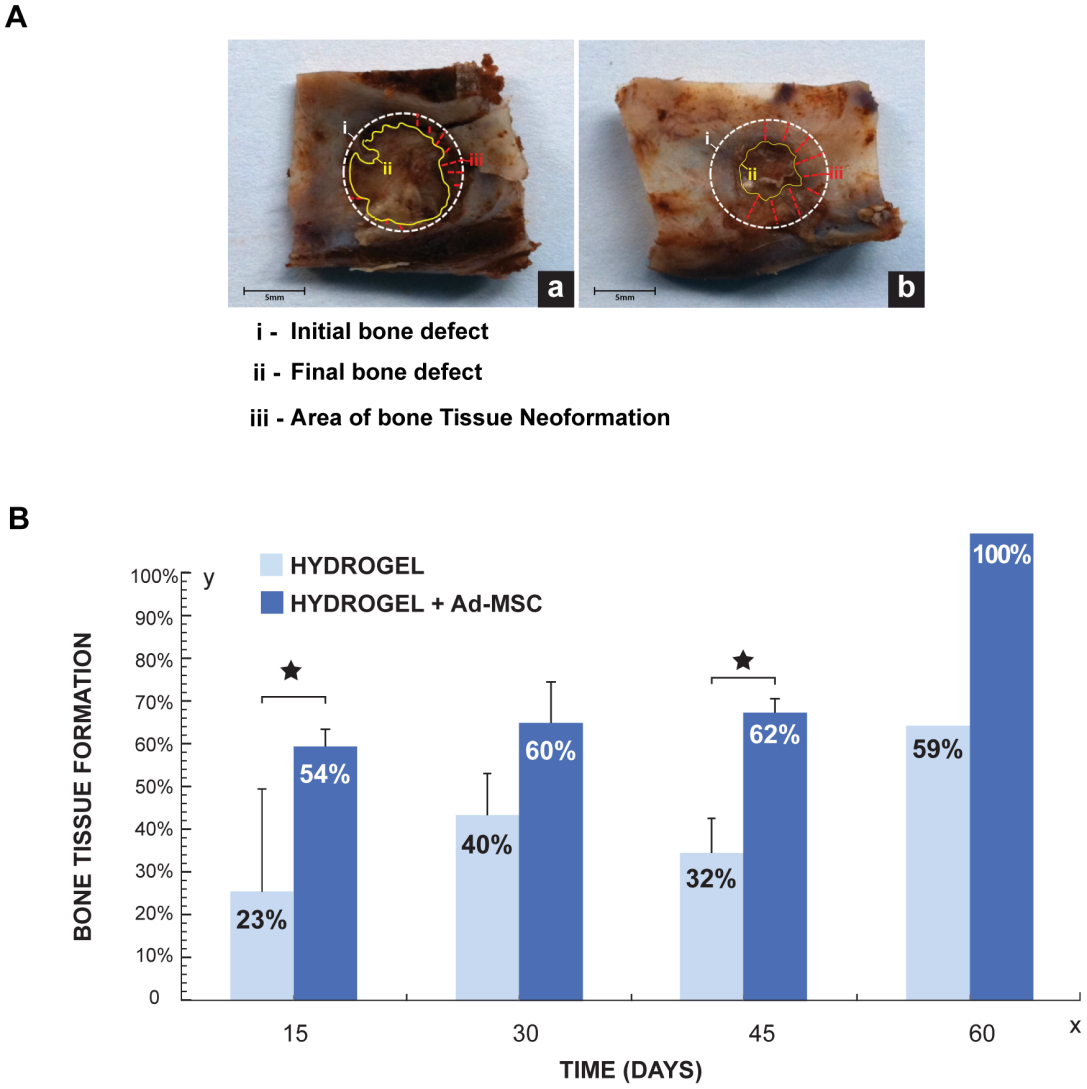
Morphometric Analysis of bone regeneration by implanting HBPHs with Ad-MSC. **A.** Surgical specimens 45 days after implantation. **a**. Hydrogel. **b.** Hydrogel with Ad-MSC. **i**, initial bone defect (white circle), **ii**, final bone defect (yellow line) and **iii**, new formed bone tissue (red lines). **B.** Percentage of newly formed bone at 15, 30, 45 (n = 4) and 60 days (n = 1), after application of Hydrogel with or without Ad-MSC.

Figure 5 shows surgical specimens 45 days after implantation with different treatments. Greater amount of newly formed bone tissue on the side treated with Ad-MSC in comparison with control side was observed 45 days after hydrogels implantation (Figure 5A, B). In Figures 5B, the percentage of new bone after different treatments is graphed. Differences in the area of new formed bone between Ad-MSC treated and control sides at 15, 30 and 45 days after hydrogels implantation are in agreement and follow the same pattern observed in the radiographic analysis (Figure 5B). Statistical analysis showed significant differences between the treated and control sides 15 and 45 days after the treatments (p≤0.05).

#### Histological Analysis

**Figure 6:**
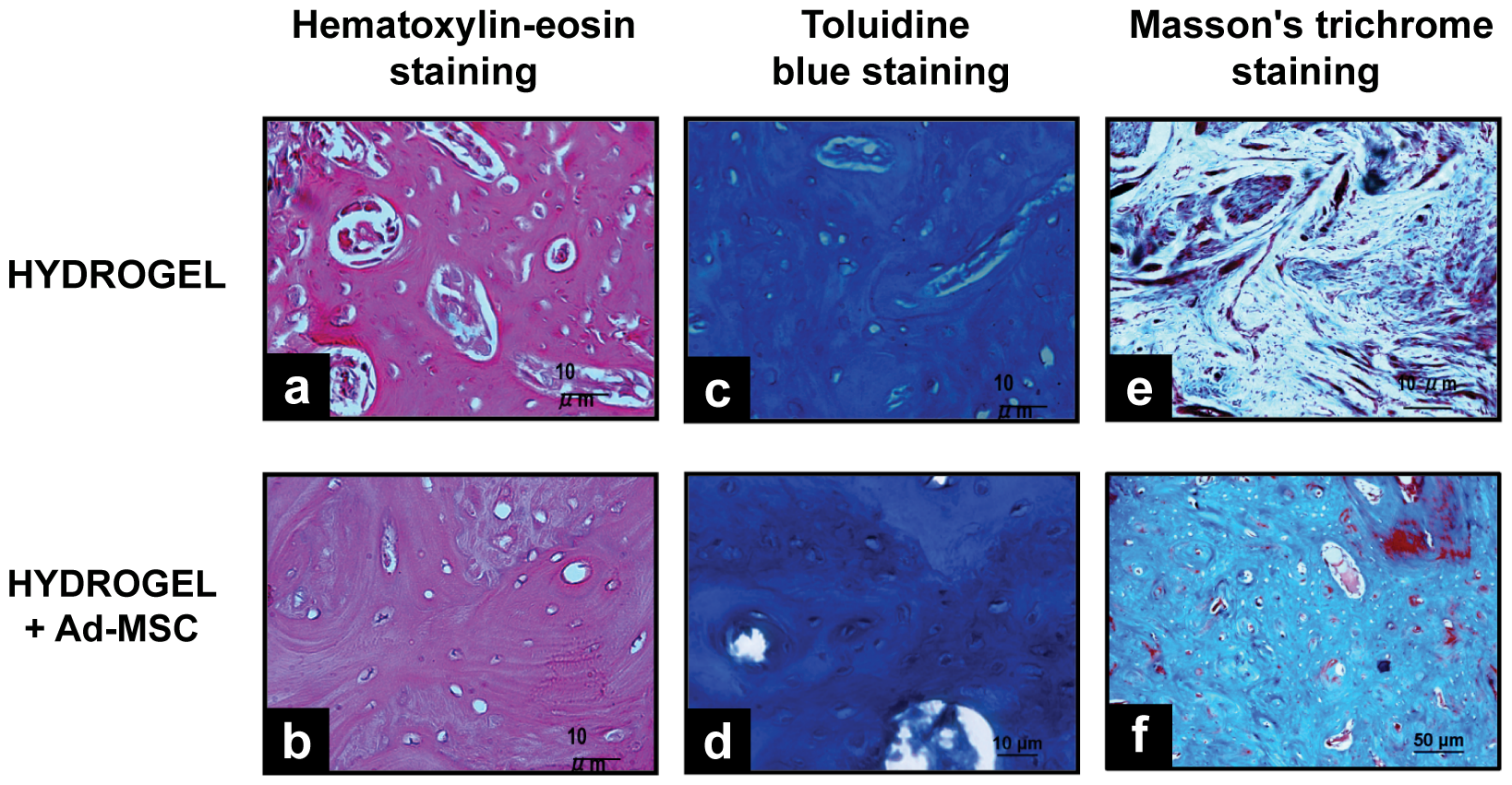
Histological Analysis of bone regeneration by implanting HBPHs with Ad-MSC. Bone defects treated with Hydrogel and Hydrogel with Ad-MSCs, 45 days after implantation. **a**, **b**, hematoxylin and eosin staining, showing a mild chronic inflammatory response. **c**, **d**, blue toluidine staining, evidencing intramembranous ossification. **e**, **f**, Masson trichrome staining, showing a better organized bone tissue and increased calcification, where hydrogels with Ad-MSC were implanted (Magnification 10×).

Figure 6, shows histologic sections of the bone regeneration zone with different treatments. Histological analysis with hematoxylin and eosin showed a mild chronic inflammatory response with and without Ad-MSC and a more organized collagen layer apposition on the side treated with Ad-MSC (Figure 6a, b). Blue toluidine staining showed that the process of ossification is intramembranous with little endochondral type ossification (Figure 6c, d). Masson trichrome staining showed that on the control side there is a lot of tissue septa arranged with disorganized mineralized matrix while in the side treated with Ad-MSC collagen fibers are organized concentrically around osteoblasts with little evidence of areas of calcification, indicating a higher grade of bone maturation (Figure 6e, f).

#### Tracking of implanted Ad-MSC

**Figure 7:**
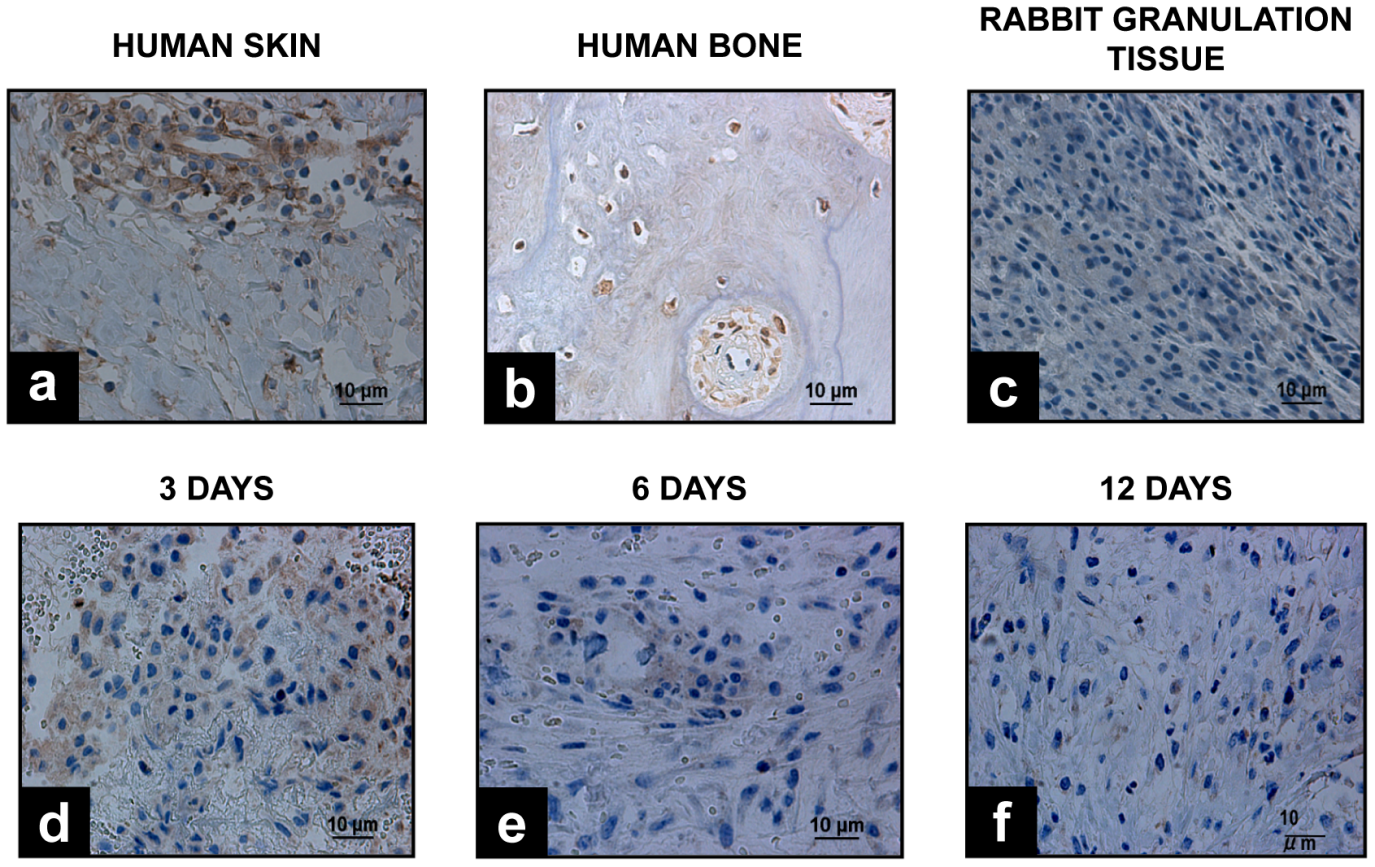
Tracking of implanted Ad-MSC. Immunohistochemical detection of positive human ß-2 microglobulin Ad-MSC. **a**, positive control, human skin. **b**, positive control, human bone. **c**, negative control, rabbit granulation tissue. Tissue regeneration zone after implantation of blood plasma hydrogel with Ad-MSCs. **d.** 3 days. **e**, 6 days. **f**, 12 days (Magnification 10×).

Implanted human cells were identified by immunohistochemistry using a specific antibody for human ß-2 microglobulin. The results show that Ad-MSC transplanted, remained at the site of injury during the first three days; however, six days after implantation, the number of cells is reduced and gradually diminishes to the point that 12 days after the implantation, no Ad-MSC were detected (Figure 7).

### Bone regeneration by implanting HBPHs with conditioned media

#### Radiographic Analysis

**Figure 8:**
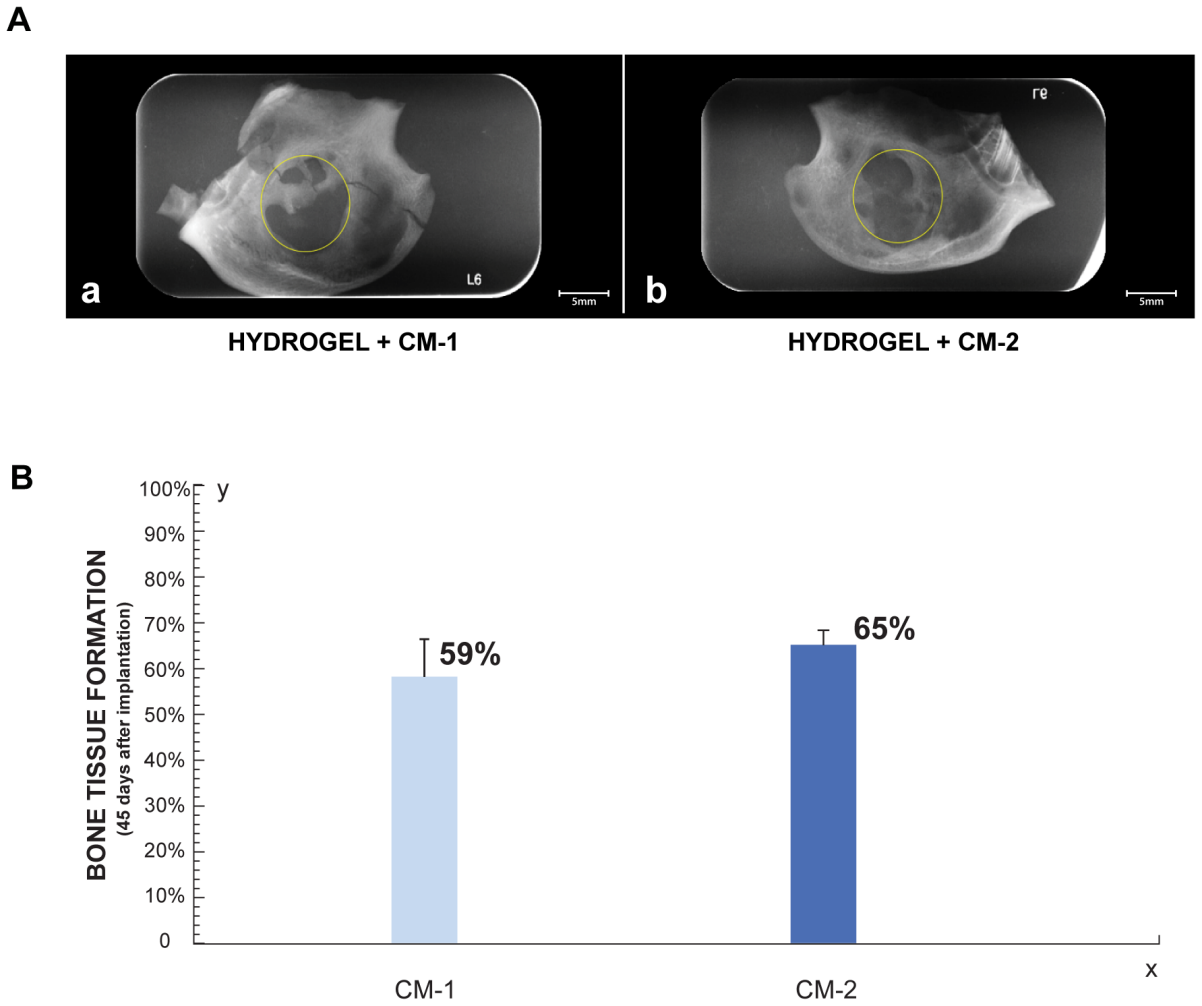
Radiographic Analysis of bone regeneration by implanting HBPHs with CM. **A** Radiographic comparison of bone defects at 45 days with CM. **a.** Bone defect treated with Hydrogel with CM-1. **b.** Bone defect treated with Hydrogel with CM-2. The circle represents the initial size of bone defect. **B.** Histogram shown the percentage of newly formed bone tissue 45 days after implantation of Hydrogel with CM (n = 3).

Radiographically, we observed the formation of new bone tissue in areas where the hydrogel with CM were implanted. In bone defects implanted with the hydrogel with CM-1 a bridge of well mineralized bone tissue is evident (Figure 8Aa) while in the defect where hydrogel with CM-2 was implanted there is greater amount of newly formed bone tissue but less mineralized (Figure 8Ab). However, there are not statistically significant differences in the amount new bone formed, with the two CM used (Figure 8B).

#### Morphometric Analysis

**Figure 9:**
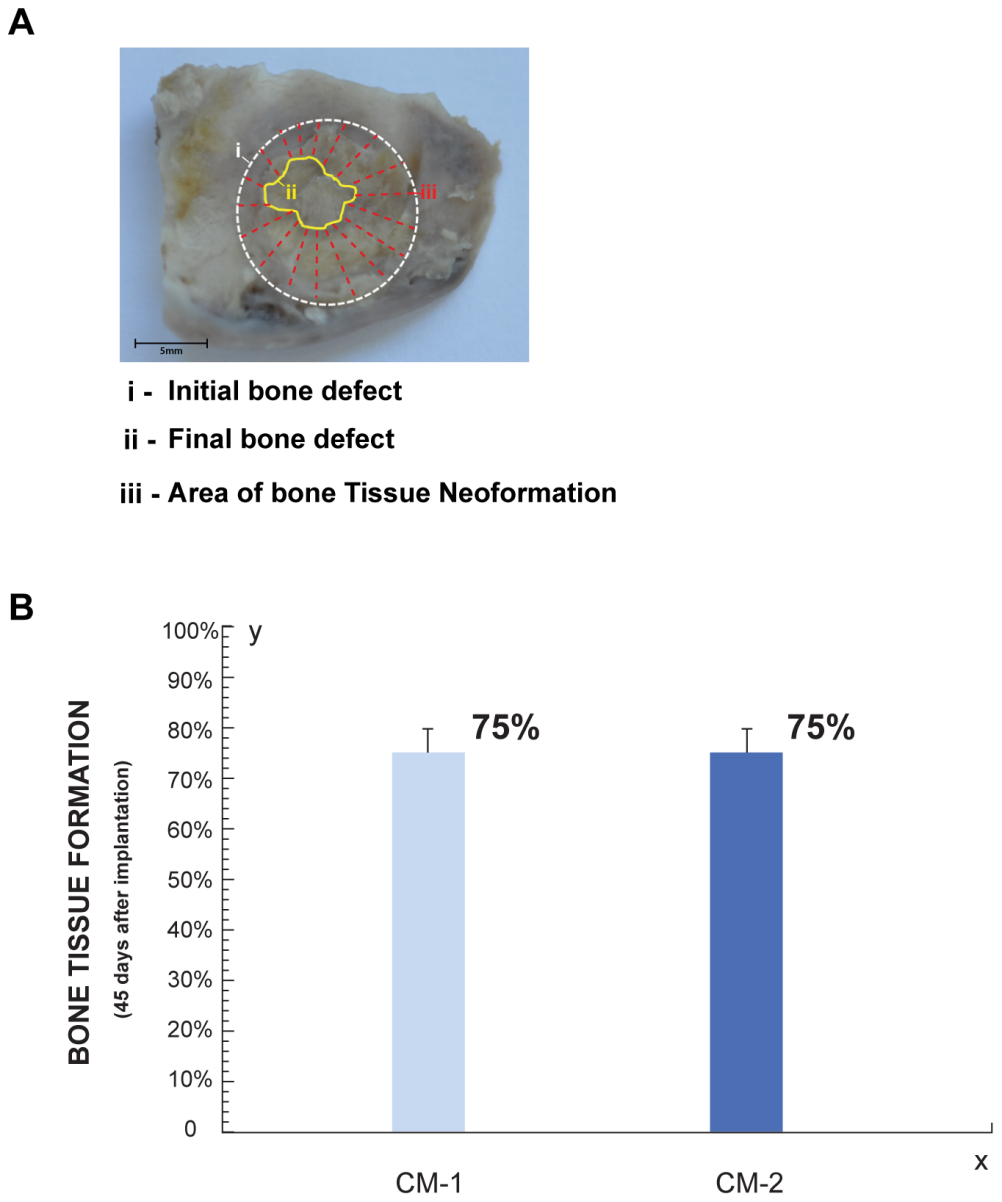
Morphometric Analysis of bone regeneration by implanting HBPHs with CM. **A.** Surgical specimens 45 days after implantation of hydrogel with CM-1. **i**, initial bone defect (white circle), **ii**, final bone defect (yellow line) and **iii**, new formed bone tissue (red lines). Surgical specimens 45 days after implantation. **B.** Histogram shown the percentage of bone neoformation 45 days after implantation of Hydrogel with CM (n = 3).

**Figure 10:**
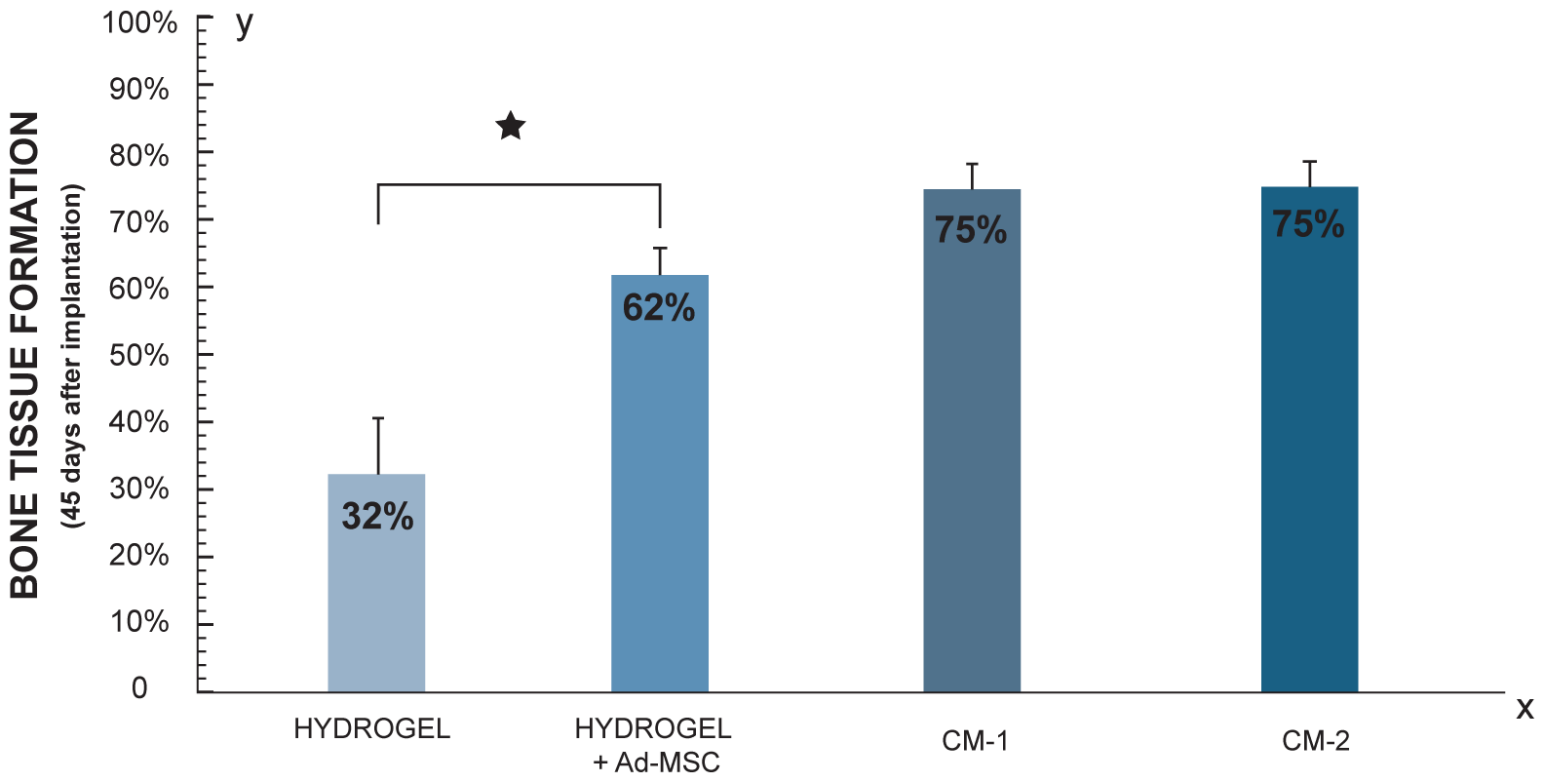
Morphometric Analysis of bone regeneration by implanting HBPHs, HBPHs with Ad- MSC and HBPHs with CM. Percentage of newly formed bone in defects treated with and without Ad-MSC (n = 4) and CM (n = 3). Bone regeneration process improves substantially where hydrogels with Ad-MSC or CM were implanted.

Newly formed bone tissue from the periphery to the center of bone defects in both sides was observed 45 days after implantation of hydrogels with CM (Figure 9A). There were not statistically significant differences between the two protein concentrations of CM used (Figure 9B). The amount of new formed bone induced by CM was comparable or even higher than that induced by the treatment with Ad-MSC (Figure 10).

#### Histological Analysis

**Figure 11:**
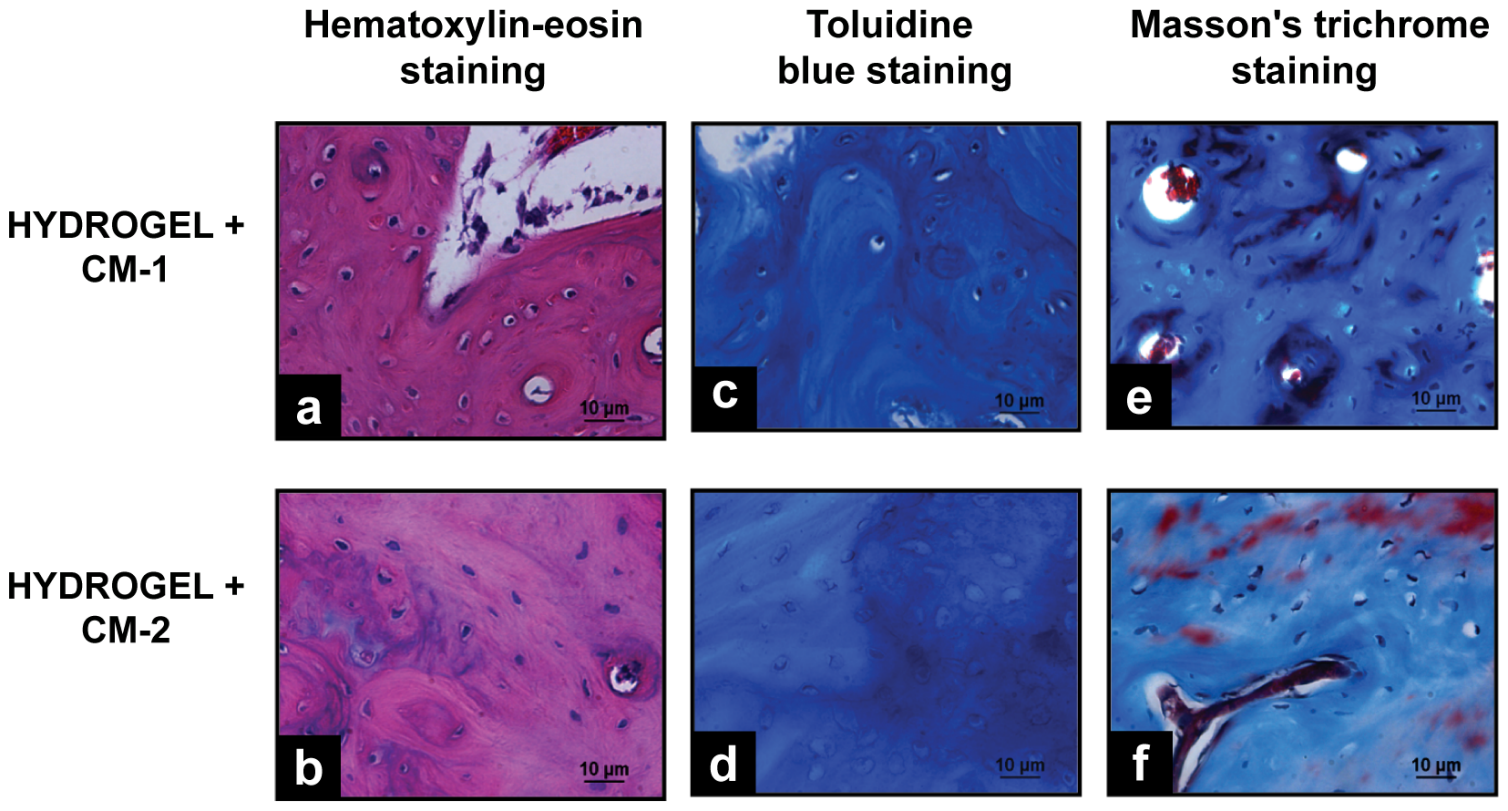
Histological Analysis of bone regeneration by implanting HBPHs with CM. Histological Analysis of bone defects treated with Hydrogel and Hydrogel with CM, 45 days after implantation. **a**, **b**, hematoxylin and eosin staining, showing a mild chronic inflammatory response. **c**, **d**, blue toluidine staining, evidencing intramembranous ossification. **e**, **f**, Masson trichrome staining, showing a the organized and calcification of bone tissue.

In the regeneration zone of bone lesions treated with CM, a mild chronic inflammatory response was observed 45 days after hydrogels implantation and as observed in the lesions treated with Ad-MSC, the tissue looks more organized than in the control side (Figure 11, a, b). The ossification was mainly intramembranous (Figure11, c, d) and with Masson trichrome staining it was evident that collagen fibers arranged concentrically around osteoblasts with red small zones indicatives of bone mineralization (Figure 11, e, f).

Histological analysis of bone regeneration in defects treated with CM and lesions treated with Ad-MSC, shows a similar type of inflammatory response (chronic mild), intramembranous ossification and similar quality and amount of newly formed bone.

## Discussion

Because of their ability to differentiate into multiple lineages, and specifically for its osteogenic potential, immunomodulatory, anti-inflammatory and antiapoptotic properties, MSC have become, the main tool of cell therapy for the treatment of diseases that functionally affects bone tissue [2], [13], [25]–[29].

Although MSC were initially isolated from bone marrow [30]–[32], now we know that the MSC can be obtained from other sources as adipose tissue [30]–[32], dental pulp tissue [31], [33], periodontal ligament [33] and periosteum [31], [32]. Currently, there is controversy in the literature on the osteogenic potential of MSCs according to their source. While some authors claim that this potential is higher in bone marrow derived MSCs compared with adipose tissue derived MSCs [6], other have shown that, skeletal muscle derived stem cells are more efficient than bone marrow-MSCs in terms of their bone regeneration capacity [7], and there are reports which found no significant differences in the regeneration of the bond defects after transplantation of MSC isolated from fat tissue, periosteum and bone marrow [8].

The bone tissue repair process is a complex cascade of biological events controlled by numerous cytokines and growth factors that provide local signals to mediate migration of osteoprogenitor cells and subsequent differentiation, cell proliferation, revascularization and production of extracellular matrix [34]. Several growth factors, such as Bone Morphogenic Proteins (BMPs) [35]–[37] platelet-derived growth factor (PDGF) [35]–[37], transforming growth factor-beta (TGF-ß) [35], [36], insulin like growth factor (IGF) [36], [38], vascular endothelial growth factor (VEGF) [35]–[38], endothelial cell growth factor (ECGF) [36], fibroblast growth factor (FGF) [34], [36], [37] and epidermal growth factor (EGF) [34] are expressed during development and bone repair, and their local availability in the wound might collaborate for faster and successful bone healing [34].

MSCs accelerate and promote new bone formation in various preclinical animal models. However, the engrafted stem cells have poor differentiation and survival rates, suggesting that the regenerative properties of these cells are exerted primarily through paracrine mechanisms [39]–[41]. Stem cells secrete a broad repertoire of trophic and immunomodulatory factors [42]. Recent preclinical studies have reported that secretomes from MSC have the potential for treating some intractable diseases as acute myocardial infarction [16], fulminant hepatic failure [43], renal failure [44], [45], ischemic stroke [46], experimental autoimmune encephalomyelitis [47] and hypoxic brain injury [48] and for the repair of soft tissues [49]. Also, has been reported that MSC-CM has a very high potential for bone regeneration, mediated by the cooperative effects of cytokines such as IGF-1, TGF-ß1 [50], [51], VEGF, angiogenin [50], HGF (Hepatocyte growth factor) [51], BMP-1 [52], IL-6, IL-3, MCP-1 (Monocyte Chemoattractant Protein-1) and MCP-3 (Monocyte Chemoattractant Protein-3) [41]. These cytokines regulate several events of osteogenesis, angiogenesis, cell migration, proliferation, and osteoblast differentiation [50]. IGF-1 induces osteoblast proliferation and migration [53]. VEGF is thought to be the main regulator of angiogenesis. VEGF also enhances survival and differentiation of endothelial cells, and as a result, it contributes to osteogenesis [54]. TGF-ß1 increases bone formation by recruiting osteoprogenitor cells and stimulating their proliferation and differentiation into osteocytes [55]. TGF-ß1 also is expressed during the development of the alveolar bone, periodontal ligament and cementum [56]. BMP-1, is an extracellular matrix (ECM) regulator that plays an important role in inducing cartilage and bone development [52] It is not a growth factor belonging to the TGF- ß family of proteins like the other BMPs, but acts as a metalloprotease cleaving the C-terminus part of procollagens I, II, and III, thus transforming them into functional ECM components [57]. Ando Y and col., locally administered conditioned medium from human mesenchymal stem cells into the distraction osteogenesis gap in a high-speed mouse model, and demonstrated that the conditioned medium promoted the formation of the new bone callus. MSC-CM recruits endogenous murine bone marrow stromal cells and endothelial cells/endothelial progenitor cells via MCP-1/-3 and IL-3/-6 signaling, respectively, and they also observed that IL-3/-6 was able to promote the osteogeneic differentiation of murine bone marrow stromal cells. These results suggest that MCP-1/-3 and IL-3/-6 act in concert to accelerate bone callus formation [41]. Recently, Inukai et al., reported induction of periodontal regeneration by endogenous cell mobilization after application of CM in a model of periodontal bone defects in dogs, supporting the hypothesis that CM improve periodontal regeneration [51].

Taken together, all these data are consistent with our results and begin to elucidate how multiple factors contained in MSC-CM cooperate to promote bone regeneration.

The results reported in this study, demonstrate that Ad-MSC significantly promote the formation of new bone in mandibular defects in rabbits. Morphometric, radiographic and histological findings show that the closure of bone defects occurs faster and better when HBPH with Ad-MSC are implanted. Newly formed bone tissue has a more organized arrangement of collagen fibers, a higher state of maturation and inorganic matrix calcification, and intramembranous ossification (Figures 6 and 11).

The fact that Ad-MSC (ß-2 microglobuline positive) can be detected after three days of transplantation, and they are not detected at all after 12 days, suggests that the survival and differentiation of Ad-MSC at the lesion site is limited. These findings are consistent with results obtained by Horie M, et al., who found that human MSC promote regeneration of articular disk in a rat model although they not persist over the injury site [14], raising some questions about the mechanisms by which MSC contribute to tissue repair after transplantation. While the answers are not completely known, accumulating evidence suggest that the therapeutic potential of MSC can be attributed not only to differentiation and integration into the injured tissue [49] but mainly to the ability to secrete soluble factors that functionally modulate the microenvironment of the host tissue in order to facilitate the endogenous process of regeneration [9], [49]. This hypothesis is supported by in vitro and in vivo studies showing that many cell types respond to paracrine signaling from MSC, regulating a large number of cellular responses, such as survival, proliferation, migration, gene expression [11], and increasing the tissue repair process in response to the application of MSC conditioned media [15], [16].

These findings supporting the MSC paracrine effect hypothesis are in agreement with the results of the present study. Morphometric, radiographic and histological data reported here demonstrate that the amount and quality of neoformed bone, repaired area, bone density, arrangement of collagen fibers, maturation and inorganic matrix calcification are very similar between Ad-MSC and CM treated groups (Figure 10).

The fact that MSC secretes therapeutic factors represents a breakthrough in regenerative medicine. In many cases, the time required to isolate and expand autologous stem cells does not allow their immediate application. The therapeutic use of CM, avoid some issues and difficulties of cell transplants. CM storage and transportation procedures are not as complex as they are for MSC. Despite the advantages of its use, CM application may not always supersede the use of MSC and it is possible that for some type of disorders MSC could be a more effective alternative. The number of known molecules mediating paracrine effect of MSC grows every day, and significantly increases the potential range of their therapeutic applications.

## Conclusions

Our results show that Ad-MSC improve bone regeneration process, and that the amount and quality of regenerated bone is similar when paracrine factors collected and applied as CM are used instead of Ad-MSC, which supports and reinforces the possibility of developing a new therapeutic strategies for the application of CM in the treatment of some bone disorders.
