# Correction: Paracrine Effect of Mesenchymal Stem Cells Derived from Human Adipose Tissue in Bone Regeneration

**DOI:** 10.1371/journal.pone.0119262

**Published:** 2015-03-04

**Authors:** The PLOS ONE Staff

There are multiple errors in Fig. 3, “Ad-MSC characterization.” Please see the corrected Fig. 3 here.

**Fig 3:**
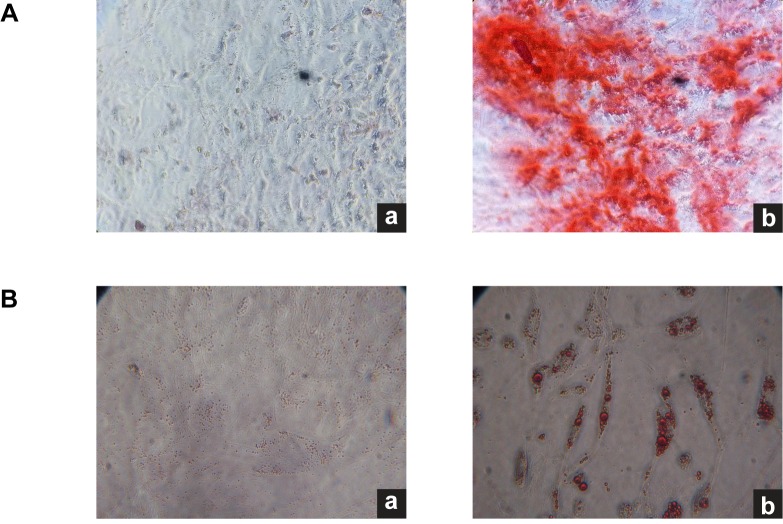
Ad-MSC characterization. A. Osteogenic differentiation of Ad-MSC. Osteogenic differentiation was evidenced by the detection of calcium deposits with Alizarin Red staining. a. Control Ad-MSCs without osteogenic induction. b. Ad-MSC cultured for 3 weeks in osteogenic differentiation medium. B. Adipogenic differentiation of Ad-MSC. Adipogenic diferentiation was evidenced by the formation of lipid vacuoles after three weeks of cultivation in adipogenic induction medium. a. Control cells without induction. b. Lipid vacuoles staining with oil red O. 10× magnification.
